# The Interfollicular Epidermis of Adult Mouse Tail Comprises Two Distinct Cell Lineages that Are Differentially Regulated by Wnt, Edaradd, and Lrig1

**DOI:** 10.1016/j.stemcr.2013.04.001

**Published:** 2013-06-04

**Authors:** Céline Gomez, Wesley Chua, Ahmad Miremadi, Sven Quist, Denis J. Headon, Fiona M. Watt

**Affiliations:** 1 Centre for Stem Cells and Regenerative Medicine, King’s College London, 28th Floor, Tower Wing, Guy’s Hospital, Great Maze Pond, London SE1 9RT, UK; 2 Norfolk and Waveney Cellular Pathology Network, The Cotman Centre, Norfolk and Norwich University Hospital, Colney Lane, Norwich NR4 7UB, UK; 3 Clinic of Dermatology and Venereology, Otto-von-Guericke University, Leipziger Strasse, 39120 Magdeburg, Germany; 4 The Roslin Institute and Royal (Dick) School of Veterinary Studies, University of Edinburgh, Midlothian, EH25 9RG, UK

## Abstract

Current models of how mouse tail interfollicular epidermis (IFE) is maintained overlook the coexistence of two distinct terminal differentiation programs: parakeratotic (scale) and orthokeratotic (interscale). Lineage tracing and clonal analysis revealed that scale and interscale are maintained by unipotent cells in the underlying basal layer, with scale progenitors dividing more rapidly than interscale progenitors. Although scales are pigmented and precisely aligned with hair follicles, melanocytes and follicles were not necessary for scale differentiation. Epidermal Wnt signaling was required for scale enlargement during development and for postnatal maintenance of scale-interscale boundaries. Loss of Edaradd inhibited ventral scale formation, whereas loss of Lrig1 led to scale enlargement and fusion. In wild-type skin, Lrig1 was not expressed in IFE but was selectively upregulated in dermal fibroblasts underlying the interscale. We conclude that the different IFE differentiation compartments are maintained by distinct stem cell populations and are regulated by epidermal and dermal signals.

## Highlights

• Tail interfollicular epidermis comprises two distinct differentiated lineages
• The lineages are maintained by unipotent progenitors that differ in cell-cycle time
• The lineages are independent of hair follicles and melanocytes
• The lineages are regulated by Wnt, Edaradd, and Lrig1

## Graphical Abstract

**Figure:**
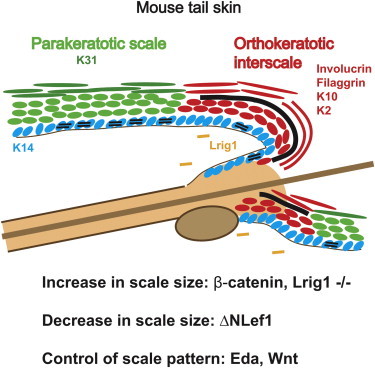

## Introduction

Mammalian epidermis is maintained by stem cells that reside in different locations, express keratin 14 (K14), and typically are anchored to the basement membrane (Arwert et al., 2012; Jensen et al., 2009). Under steady-state conditions, epidermal stem cells only give rise to the differentiated cells that are appropriate for their location, but when the tissue is injured or subjected to genetic modification, they can give rise to any differentiated epidermal lineage (Arwert et al., 2012; Jensen et al., 2009).

Lineage tracing using a ubiquitous inducible promoter suggests that mouse interfollicular epidermis (IFE) is maintained by a single population of cells that upon division can produce two basal cells, two differentiated cells, or one of each (Clayton et al., 2007; Doupé et al., 2010). In contrast, combined lineage tracing using K14CreER and CreER driven by a fragment of the Involucrin promoter (Inv) suggests that slow-cycling stem cells give rise to more rapidly cycling committed progenitors that subsequently undergo terminal differentiation (Mascré et al., 2012). Such studies rely on clonal analysis of whole mounts of tail epidermis (Braun et al., 2003), but overlook the fact that there are two programs of terminal differentiation (orthokeratotic and parakeratotic) within tail IFE. This raises the question as to whether the basal layer of tail IFE contains cells with uni- or bipotent differentiation capacity.

In tail epidermis, the hair follicles (HFs) are arranged in groups of three (triplets) in staggered rows (Braun et al., 2003). The IFE adjacent to the HFs, known as the interscale IFE, undergoes orthokeratotic differentiation, culminating in the formation of a granular layer in the outermost viable cell layers and loss of nuclei in the cornified, dead cell layers that cover the surface of the skin. Orthokeratotic differentiation is also characteristic of dorsal IFE. In contrast, tail IFE that is not adjacent to the HFs, known as the scale IFE, undergoes parakeratotic differentiation characterized by the lack of a granular layer and retention of nuclei in the cornified layers. Scales, like HFs, are regularly spaced and arranged in rows that form rings around the tail. The infundibulum of each HF connects with the interscale IFE while the hair shafts overlie the scales.

At birth, the entire tail epidermis is orthokeratotic (Didierjean et al., 1983; Schweizer and Marks, 1977). Scale formation is first detected 9 days after birth (Didierjean et al., 1983; Schweizer and Marks, 1977). Little is known about the mechanisms of scale induction and maintenance, although topical application of vitamin A to adult tail skin reversibly converts scales into interscales (Schweizer et al., 1987).

In this study, we examined whether the two programs of tail IFE differentiation arise from a common, bipotent population of cells in the basal layer, and identified signaling pathways that regulate scale formation and maintenance.

## Results

### Development of Scale and Interscale IFE in Postnatal Tail Epidermis

**Figure 1:**
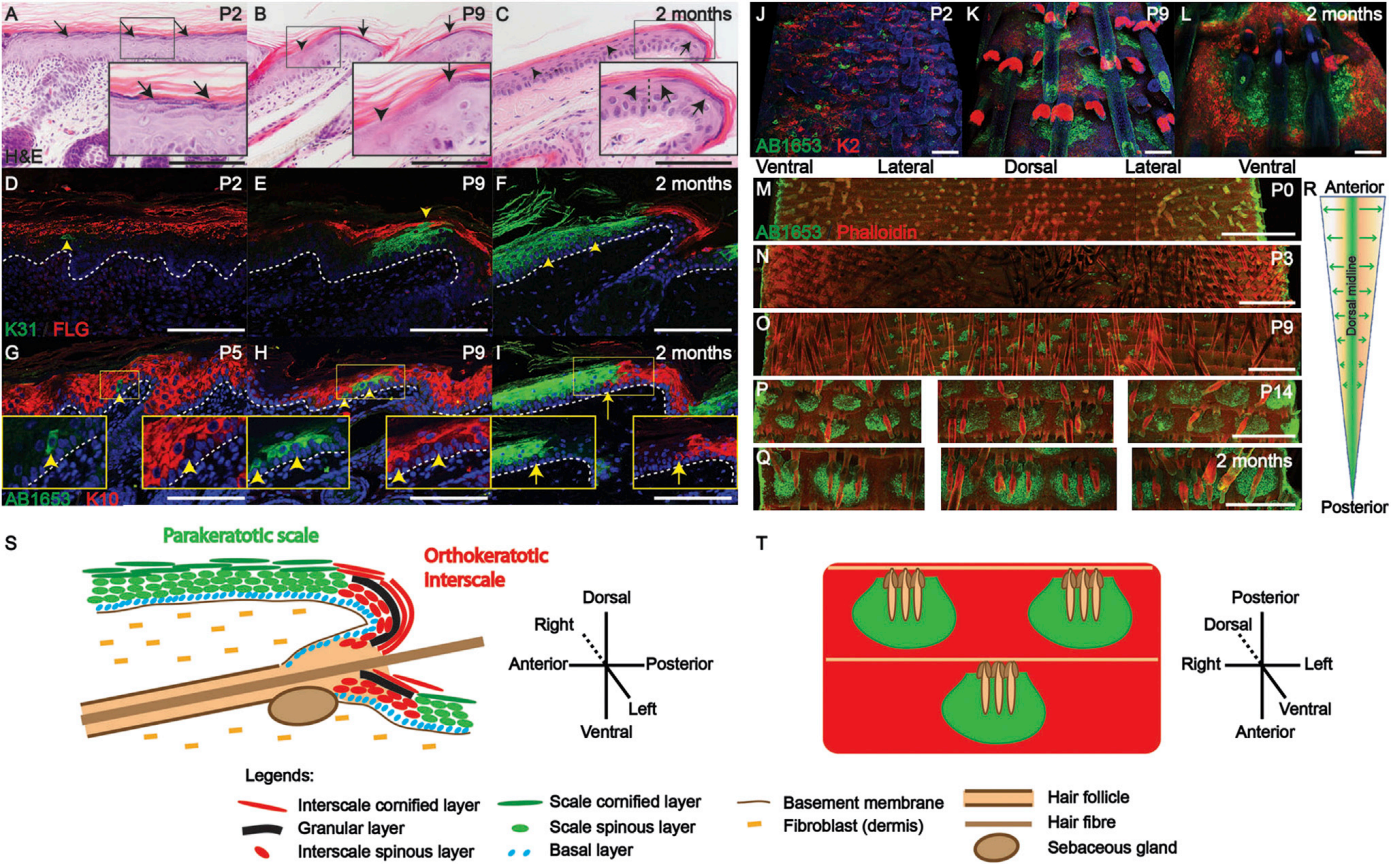
Differentiation of Scale and Interscale IFE (A–I) Sagittal sections of WT tail skin. (A–C) Hematoxylin and eosin (H&E) staining. (D–F) Immunostaining for FLG (red) and K31 (green). (G–I) Immunostaining for K10 (red) and AB1653 (green). Insets are single-stained, enlarged views of the boxed areas. Interscale (arrows); scale (arrowheads); DAPI counterstain (blue); dermo-epidermal junction (white dashed lines). (J–Q) Whole mounts of WT tail epidermis. (J–L) Immunostaining for K2 (red) and AB1653 (green). (M–Q) AB1653 immunostaining (green) with phalloidin (red) counterstain. Images were obtained from the anterior part of the tail. (R) Scales are induced along the dorsal midline at P3 and spread laterally to reach the ventral midline by P14. (S and T) Schematics showing sagittal (S) and whole-mount (T) views of scale and interscale IFE in 2-month-old mice. In all panels, scales are green and interscales are red. Scale bars, 100 μm (A–L) and 500 μm (M–Q). See also Figure S1.

To determine when scale and interscale IFE becomes specified, we labeled postnatal tail epidermis with antibodies to filaggrin (FLG), keratin 10 (K10), and keratin 2 (K2), three markers of orthokeratotic differentiation (Brown and McLean, 2012; Moll et al., 2008). At birth, tail IFE exhibited a continuous granular layer and expressed FLG in the upper spinous layers (Figures 1A and 1D). K10 and K2 were expressed by cells in all of the underlying suprabasal layers (Figure 1G; Figure S1A available online). At postnatal day 9 (P9), there was focal loss of the granular layer (Figures 1B and 1E), with a corresponding loss of K10 and K2 in the underlying suprabasal cells (Figures 1H and S1B), marking the onset of parakeratosis. At 8 weeks, the alternating pattern of parakeratotic scales and orthokeratotic interscales was fully developed (Figures 1C, 1F, 1I, and S1C).

We labeled scale IFE with anti-K31, which in other body sites is confined to HFs (Langbein et al., 1999), and with AB1653, which recognizes caspase 14 and a range of scale proteins, including keratins (Figures S1D–S1G and data not shown). There was complete coexpression of K31 and AB1653 antigens in scale cells at all stages of development (Figures S1H–S1J). Single cells undergoing scale differentiation (K31+, AB1653+, K10−, and K2−) appeared in the suprabasal layers between P0 and P3 (Figures 1D, S1K, and S1L; Didierjean et al., 1983). These became cell clusters that expanded laterally and vertically, until by P14 they extended to the outermost, cornified layers (Figures 1E, 1G, 1H, and S1M). Scale and interscale markers were never coexpressed (Figures 1D–1L).

Scale induction was initially restricted to the tail dorsal midline (Figures 1M, 1N, and S1N–S1O) and then expanded laterally, reaching the ventral midline by P14 (Figures 1O, 1P, and 1R). Individual scales first formed in front of each HF triplet, gradually enlarging to reach their final size by 8 weeks (Figures 1Q and S1P). In adult tail epidermis, involucrin expression is largely confined to interscale IFE (Figure S1Q); almost all involucrin-positive cells are suprabasal and the subset of basal cells that express involucrin lie at the scale-interscale boundary (López-Rovira et al., 2005). This is also the case for transgenes expressed under the control of the Inv promoter (Carroll et al., 1995; Lapouge et al., 2011; Figures S1R and S1S), indicating that InvCreER (Mascré et al., 2012) will label committed progenitors of interscale and not scale. Figures 1S and 1T illustrate schematically the pattern of scale and interscale IFE differentiation.

### Scales Are Founded by Unipotent Progenitors

**Figure 2:**
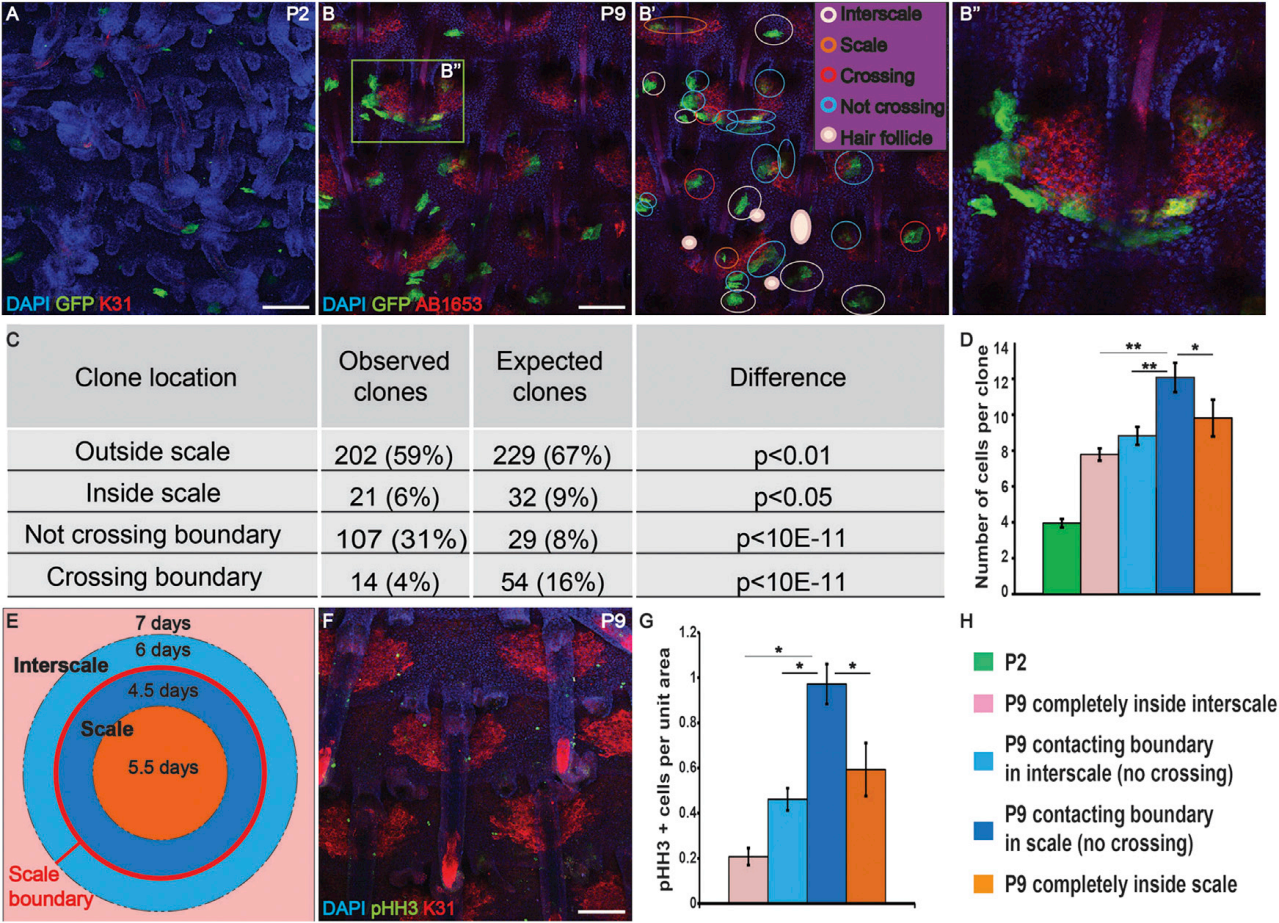
Clonal Analysis of Scale and Interscale Epidermis (A and B) Tail epidermal whole mounts of K14CreER × CagcatGFP mice immunostained for GFP and K31 at the stages indicated. (B′) Analysis of GFP-positive clones in (B). GFP clones: interscale (pink), scale (orange), encompassing scale and interscale (red), and bordering scale without crossing (blue) or HF (solid pink). (B″) Enlargement of the boxed region in (B). (C and D) Quantitation of observed and expected clone types (C) and number of cells per clone (D). t test, ^*^p < 0.05, ^**^p < 0.001. Error bars represent SEM. (E) Rates of cell division in different areas. (F) Immunostaining for K31 (red) and pHH3 (green) of WT P9 tail epidermis. (G) Spatial distribution of pHH3 positive cells. z test, ^*^p < 0.05. Error bars represent SD. (H) Legends and color code for (D), (E), and (G). Scale bars, 100 μm. See also Figure S2.

To investigate whether scale and interscale differentiated cells arise from a common progenitor population, we used K14CreER to induce GFP expression in single cells in the basal layer of CAG-CAT-eGFP mice upon topical application of 4-hydroxy-tamoxifen (4OHT) at P0. By P2, clones of two to four GFP-positive cells were detected throughout the IFE, widely separated by unlabeled cells (Figures 2A and S2A–S2D). We scored the location of individual clones in P9 tail epidermal whole mounts (Figures 2B and 2B′).

If scale and interscale are founded by bipotential basal cells (Mascré et al., 2012), cross-boundary clones should be detected. In contrast, if they are founded by unipotent basal cells, clones should fall in either the scale or interscale area and should not cross boundaries. To calculate the expected number of clones of each type if basal cells are bipotential, we determined that scale accounts for 20% of the total surface area of tail IFE at P9, and assumed that the number of clones in a given area is proportional to its size (Supplemental Experimental Procedures; Figures S2E–S2G). Only 4% of the clones examined (n = 344) crossed scale boundaries, which is far fewer than predicted by the bipotent progenitor model (p < 10^−11^; Figure 2C). We found that 31% of the clones had edges that coincided with, but did not cross, boundaries (Figure 2C) and were often elongated in shape, suggesting that they were actively excluded from scales (Figure 2B″). The rest of the clones were distributed completely inside scale (6%) or interscale (59%) away from the boundaries (Figure 2C). The observed frequency of all clone types was significantly different from the expected frequency (Figure 2C). Clones in adult mice remained largely restricted to one compartment (Figures S2H–S2K).

Clones within scales were significantly larger than interscale clones, and scale clones that contacted the interscale boundary were larger than those that were fully inside the scales (Figures 2D and 2H). Knowing that clones took 7 days to form (from P2 to P9), we calculated the average cell-cycle time in different regions of tail IFE (Supplemental Experimental Procedures). Cells in scales at the interscale boundary divided every 4.5 days, i.e., faster than cells in the center of scales (5.5 days) and in interscales (6–7 days; Figures 2E and 2H). We confirmed this by scoring the number of phospho-histone H3 (pHH3)-positive cells (Figures 2F–2H). Thus, scales expand by proliferating more rapidly than interscale regions, which explains the contour of interscale clones at scale boundaries (Figure 2B″).

Since we never observed scales that were uniformly positive for GFP, each scale must be polyclonal in origin. We never observed clones extending between HF and IFE, indicating that scales do not originate from HF stem cells (Arwert et al., 2012).

### Scale and Interscale Patterning Is Independent of Melanocytes

**Figure 3:**
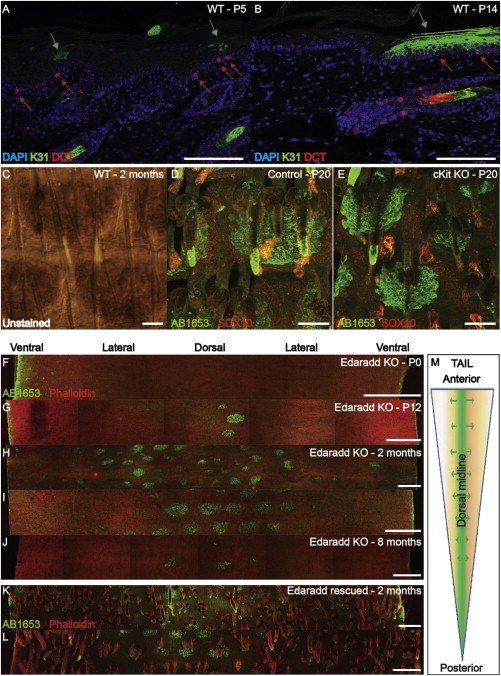
Scale Formation Occurs in the Absence of Melanocytes and HF (A and B) Immunostaining of WT tail skin sagittal sections with DCT and K31 antibodies. Melanocytes (red arrows); scale differentiation (green arrows); DAPI counterstain (blue). (C) Bright-field view of WT 2-month-old tail epidermal whole mount showing scale pigmentation. (D and E) Immunostaining for AB1653 (green) and SOX10 (red) in P20 epidermal whole mount of ckit knockout (E) and WT littermate (D) mice. (F–J) *Edaradd* knockout tail epidermis labeled with AB1653 (green) and phalloidin (red). (F) n = 6 mice, (G) n = 4, (H and I) n = 10, (J) n = 3. (K and L) Two-month-old *Edaradd* knockout rescued with K14::Edaradd transgene, labeled with AB1653 (green) and phalloidin (red) (n = 5). (M) Diagram of scale induction in *Edaradd* knockout. Scale bars, 100 μm (A–E) and 500 μm (F–L). See also Figure S3.

The spatiotemporal activation of scale differentiation from the dorsal to the ventral surface of the tail is reminiscent of the migration path taken by neural crest cells early during development (Sommer, 2011). Neural crest cells are melanocyte precursors, and one striking characteristic of scale IFE is that it contains resident pigmented melanocytes (Figure 3C; Didierjean et al., 1983; Schweizer and Marks, 1977). Double-label immunostaining for dopachrome tautomerase (DCT), a melanocyte and melanoblast marker (Tsukamoto et al., 1992), and K31 showed that melanocytes were located in the basal layer of the IFE prior to the onset of scale differentiation (Figures 3A and 3B).

In *cKit* mutant mice, neural crest cell migration is impaired and the skin lacks melanocytes (Cable et al., 1995), as confirmed by lack of Sox10 expression (Figures 3D and 3E). Scale formation was normal in *cKit* mutant mice (Figures 3D and 3E), although scales tended to be smaller because the mice were developmentally delayed.

### Scale and Interscale Patterning Is Independent of HFs

Mice with null mutations in the ectodysplasin pathway have hairless tails (Sundberg, 1994). In *Edaradd* knockout mutants, a transient condensation of epidermal cells (Figures S3A and S3B), which express P-cadherin but lack the early HF marker Sox9 (Nowak et al., 2008; Figures S3C and S3D), occurs at P0 but disappears by P2 (Schmidt-Ullrich et al., 2006).

Scale induction in *Edaradd* knockout mutants was initiated at P10, significantly later than in wild-type (WT) mice (Figures 3F and 3G). The number and size of the scales were variable and scales formed only on the dorsal surface (Figures 3G–J, and 3M). Nevertheless, the pattern of scale induction was partly conserved (Figure 3I). Those scales that formed did so normally (Figures S3E–S3H).

We rescued HF formation by crossing *Edaradd* knockouts with K14*Edaradd* transgenic mice (Figures 3K and 3L). In contrast, scale induction was not rescued and no scales formed on the ventral midline (Figures 3K and 3L). We conclude that HF and scale induction can be uncoupled, and identify a role for *Edaradd* in the ventral induction of scales.

### Wnt Signaling Controls Scale Shape and Maintenance

**Figure 4:**
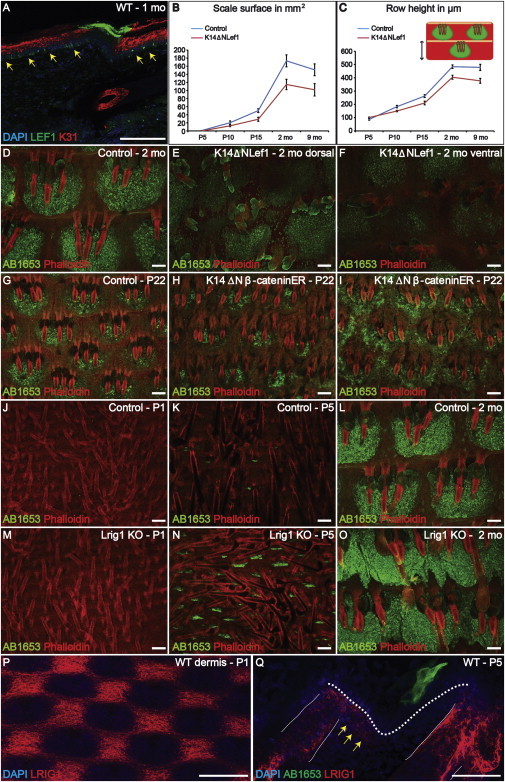
Wnt and Lrig1 Signaling Controls Scale Borders (A) WT tail skin labeled with LEF1 and K31 antibodies and DAPI counterstain. (B and C) Differences in scale size (B) and distance between rows of HF (C) in K14ΔNLef1 and control littermates. Error bars represent SEM. (D–O) Immunostaining with AB1653 (green) and phalloidin counterstain (red) of tail epidermal whole mounts: (D) 2-month-old control and (E–F) K14ΔNLef1 littermates (dorsal [E] and ventral [F] views), (G) control and (H–I) 4OHT-treated K14ΔNβ-cateninER transgenics, Lrig1 knockouts (M–O), and WT littermates (J–L). (P) LRIG1 immunostaining of P1 WT tail dermis. (Q) AB1653 and LRIG1 immunostaining of WT P5 tail skin. Epidermal basal layer (dotted lines); HF (solid lines); dermal LRIG1 expression (yellow arrows). Scale bars, 100 μm. See also Figure S4.

The Wnt pathway regulates lineage selection in postnatal epidermis (Watt and Collins, 2008). Lef1 was upregulated in the basal layer of adult tail scale, but not interscale or dorsal IFE (Figure 4A, arrows; Niemann et al., 2002). This led us to examine K14ΔNLef1 mice, in which epidermal Wnt signaling is inhibited by dominant-negative Lef1 (Niemann et al., 2002). Initiation of scale formation was delayed to P4, and K14ΔNLef1 scales were smaller than WT scales (Figure 4B). This correlated with a decrease in the distance between HF rows and, since tail length was normal, an increase in the number of rows of HF (Figure 4C).

Although the first postnatal hair cycle in K14ΔNLef1 mice is normal, HFs subsequently convert into IFE cysts with ectopic sebocytes (Niemann et al., 2002). From 2 months (Figures 4D–4F and S4A–S4D), ∼ 10% of scales were irregular in shape and fused or absent, and by 9 months up to 25% of scales were lost (Figures S4A–S4D). PHH3 labeling in 4-month-old K14ΔNLef1 epidermis revealed a selective increase in interscale proliferation relative to WT (Figure S4E).

We also used a gain-of-function approach in which a constitutively active form of β-catenin (K14ΔNβ-cateninER) was induced by topical application of 4OHT for 3 weeks to P2 and adult skin. This led not only to induction of ectopic HF (Lo Celso et al., 2004; Silva-Vargas et al., 2005) but also to expansion of scales and loss of definition of scale-interscale boundaries. In some cases, adjacent scales merged with one another (Figures 4G–4I and S4F–S4H). The changes in scales did not correspond to sites of ectopic HF formation in the IFE (Figures S4I and S4J).

### Regulation of Scale Size by Lrig1

Lrig1, a negative regulator of EGF receptor signaling, is a marker of HF junctional zone stem cells (Jensen et al., 2009). The Lrig1-positive population does not contribute to the IFE (Jensen et al., 2009), but loss of Lrig1 results in tail IFE hyperproliferation and increased pigmentation (Jensen et al., 2009). Scales were induced with the same timing in Lrig1 knockout and WT mice (Figures 4J, 4K, 4M, and 4N). However, scales were larger (Figures 4K and 4N) and were fused laterally (Figures 4L and 4O), consistent with the finding that scale epidermis expands by more rapid proliferation compared with interscale IFE (Figure 2E).

Lrig1 is not differentially expressed in WT scale and interscale IFE (Jensen et al., 2009). However, at P1, prior to scale formation, Lrig1 was expressed in the upper dermis in a pattern corresponding to the presumptive interscale (Figures 4P and 4Q). Thus, Lrig1 may control the size of the scale compartment by differential expression in the dermis and serve as a template for defining future scale locations.

## Discussion

We have shown that scale and interscale IFE is maintained by discrete unipotent populations of basal cells. Each population must include stem cells because both differentiation programs are maintained throughout adult life independently of HF stem cells (Jones et al., 2007). This forces a reappraisal of earlier lineage tracing studies, in particular those that used InvCreER as a marker of IFE progenitors. In tail IFE, almost all involucrin-positive cells are suprabasal and involucrin is only expressed in a subset of basal cells at the scale-interscale boundary (López-Rovira et al., 2005; Figures S1Q–S1S). Therefore, InvCreER (Mascré et al., 2012) will label committed progenitors of only one lineage.

Our observation of different rates of proliferation in the basal cell layer of tail IFE is consistent with earlier findings (Mascré et al., 2012). However, this difference is not due to slow-cycling stem cells giving rise to rapidly cycling committed progenitors (Mascré et al., 2012). Instead, scale cells maintain a faster rate of division than interscale cells in adult tail skin under homeostatic conditions (Figure S4E). It is interesting that we found basal-layer involucrin-positive cells in the region of most rapid proliferation, since this may prevent unlimited scale expansion and is consistent with studies in human IFE (Watt et al., 1987). Our data call into question the conclusion that differences in clone size within tail IFE are entirely due to chance (Clayton et al., 2007; Doupé et al., 2010). It would be interesting to initiate lineage tracing in adult mice to document the behavior of the two lineages during epidermal homeostasis.

Examination of *cKit* and *Edaradd* mutant mice showed that scale formation was regulated independently of melanocytes and HF. Rescue of *Edaradd* expression in the epidermis restored HF (albeit with altered patterning) but not ventral scale formation. The ligand *Eda* and its receptor, *Edar*, are differently patterned in mouse back epidermis (Laurikkala et al., 2002). This suggests that patterned expression of *Edaradd* in cells that do not express K14 is required for induction of ventral scales. During wound healing, epidermal stem cells can adopt new locations that differ from their location in undamaged epidermis (Arwert et al., 2012), and therefore it would be interesting to discover whether wounding *Edaradd* mice influences the location or behavior of the tail IFE populations.

Epidermal Wnt signaling regulated scale formation and maintenance. Inhibition of *Lef1* resulted in abnormally small scales, correlating with a selective increase in interscale proliferation. Conversely, activation of β-catenin resulted in scale expansion and blurring of interscale boundaries. It would be interesting to perform clonal lineage tracing in these mice and other mice with disturbed scale patterning to gain further insights into the fate of scale and interscale progenitors.

We found that the scale compartment expands via proliferation at its edges. Consistent with this finding, loss of Lrig1, which leads to IFE hyperproliferation (Jensen et al., 2009), resulted in expansion and lateral fusion of scales. Since the IFE does not express detectable levels of Lrig1, the patterned expression of Lrig1 in the dermis underlying future interscales may regulate proliferation in the overlying epidermis. Lrig1 and Lef1/β-catenin signaling may act in competition to regulate the pool of scale stem and progenitor cells, with Lrig1 keeping the pool small and Lef1/β-catenin expanding it. Interscale expansion also occurs upon loss of the Notch ligand Dll1 (Estrach et al., 2008) or epidermal deletion of β1 integrin (López-Rovira et al., 2005), again corresponding to increased proliferation. Together, these results reveal an interacting network of signaling pathways that act in both the epidermis and dermis to differentially regulate programs of terminal differentiation within tail IFE.

## Experimental Procedures

### Mice

Procedures were performed under a UK Government Home Office license. The WT mice were C57 Bl/6 × CBA F1. *K14-creER* (Jax strain, stock number 005107), *CAG-CAT-eGFP* (Kawamoto et al., 2000), *W/ckit mutant* (Cable et al., 1995; a gift from I.J. Jackson), *crinkled/Edaradd*, *crinkled::K14::Edaradd* (Heath et al., 2009), *K14ΔN-β-cateninER* (line D2; Lo Celso et al., 2004), *K14ΔNLef1* (Niemann et al., 2002), *Lrig1* null (Suzuki et al., 2002), and *Caspase14* null (Denecker et al., 2007; a kind gift from W. Declercq) mice were described previously. InveGFPRac1QL (line 7596) mice express activated Rac1eGFP via the Inv promoter (Carroll et al., 1995; Lapouge et al., 2011).

Two-month-old *K14ΔN-β-cateninER* mice (n = 8) and WT littermates (n = 7) were treated with 1.5 mg 4OHT in 100 μl acetone three times a week for 3 weeks. P0 *K14ΔN-β-cateninER* pups (two litters, 17 pups total) were treated with increasing doses of 4OHT (225 μg to 1.125 mg) on the tail three times a week for 3 weeks.

For lineage tracing, K14-CreER × CAG-CAT-eGFP mice were treated at P0 with 0.1 mg 4OHT in 10 μl acetone. The tail skin of three pups per litter (n = 7) was harvested 2 days later and the remaining pups were allowed to develop to P9 (n = 19), P15 (n = 3), P31 (n = 3), and P100 (n = 3) prior to collection of tail skin.

### Immunohistochemistry

Details regarding antibodies, antigen retrieval, and whole-mount preparation are given in the Supplemental Experimental Procedures. Skin for sectioning was fixed in 4% formaldehyde and embedded in paraffin. The epidermal whole-mount procedure was described previously (Braun et al., 2003).

### Image Capture and Analysis

Microscopy was carried out using a Leica SP5 confocal microscope and LASAF software. Images and z stacks were obtained using 10× dry 0.3 na and 20× dry 0.7 na objectives.

K2/AB1653 images for Figures 1J–1L were deconvolved using Autodeblur software and three-dimensional views were obtained after deconvolution using Imaris software. Images were optimized globally for brightness, contrast, and color balance using Photoshop CS4 (Microsoft) and assembled into figures with Adobe Illustrator CS4 (Microsoft). Measurements of scale surfaces were performed using ImageJ software.

## Supplemental Information

Document S1. Figures S1–S4 and Supplemental Experimental Procedures
