# Supplementary material for: The Interfollicular Epidermis of Adult Mouse Tail Comprises Two Distinct Cell Lineages that Are Differentially Regulated by Wnt, Edaradd, and Lrig1
Source: Stem Cell Reports. 2013 Jun 4;1(1):19–27. doi: 10.1016/j.stemcr.2013.04.001 (PMC3757744; doi:10.1016/j.stemcr.2013.04.001)
Supplement: Document S1. Figures S1–S4 and Supplemental Experimental Procedures [file mmc1.pdf]

Stem Cell Reports, Volume *1*

Supplemental Information

The Interfollicular Epidermis of Adult Mouse Tail Comprises Two Distinct Cell Lineages that Are Differentially Regulated by Wnt, Edaradd, and Lrig1

Céline Gomez, Wesley Chua, Ahmad Miremadi, Sven Quist, Denis J. Headon, and Fiona M. Watt

Inventory of Supplemental Information

Supplemental Figure legends

Supplemental Materials and Methods

Figure S1, relates to Figure 1.

Figure S2, relates to Figure 2.

Figure S3, relates to Figure 3.

Figure S4, relates to Figure 4.

Figure S1: (**A-C**) Immunostaining of tail skin sagittal sections for keratin 2 (K2) at P2 (**A**), P9 (**B**) and 2 months (**C**); (**D-G**) Immunostaining of control (**D, F**) and caspase 14 knock-out (**E, G**) tail epidermal wholemounts with AB1653 (**D, E**) and an anti-caspase 14 antibody kindly provided by Wim Declercq (**F, G**). (**H-M**) Immunostaining of tail skin sagittal sections for K31 and AB1653 at the stages indicated. Note colocalisation of K31 and AB1653 antigen in scales (**H-J**). (**N-Q**) Immunostaining of wild-type tail epidermal wholemounts for K31 (**N-P**) and involucrin (INV) (**Q**). (**R-S**). InveGFPRac1QL (line 7596) mouse tail skin section (**R**) and epidermal wholemount (**S**) immunostained for GFP. Scale bars = 100 μm (**A-C, H-M, R**), 500 μm (**D-G, N-Q,S**).

Figure S2: (**A-D**) K14CreER-cagcatGFP tail epidermal wholemounts immunostained for GFP (green) and K31 (red) at P2, 2 days after 4OHT application. (**E-G**) Calculation of expected number of clones based on random distribution within scale and interscale IFE. See Supplemental Materials and Methods for calculations. (**H-J**) K14CreER-cagcatGFP tail epidermal wholemounts immunostained for GFP (green) and AB1653 (red) at P15 (**H**), P31 (**I**) and P100 (**J**). Inserts are enlarged views of boxed areas. (**K**) Total number of clones scored (left column) and number of clones crossing scale boundaries (right column) at P15, P31 and P100. Scale bars = 100 μm.

Figure S3: (**A-D**) Staining of P0 *Edaradd* heterozygous control (**A, C**) and P0 *Edaradd* knock-out mutant (**B, D**) tail epidermal wholemounts with DAPI (**A, B**; blue), P-cadherin (**C, D**; green) and SOX9 (**C, D**; red). (**E-H**) Immunostaining for FLG and K31 in sagittal sections of *Edaradd* knock-out and wild type tail skin at the ages indicated. Scale bars = 100 μm.

Figure S4: (**A-C, F-H**) Immunostaining with AB1653 (green) and phalloidin counterstain (red) of tail epidermal wholemounts: (**A**) 9 month old control, (**B-C**) 9 month old K14ΔNLef1 littermates (B: dorsal; C: ventral views), (**F**) 3 month old control, (**G-H**) 4OHT treated 3 month old K14ΔNβ-cateninER transgenic mice. (**D**) Quantitation of scale defects observed in the dorsal and ventral regions of 2 and 9 month old K14ΔNLef1 mice and control littermates. n = number of scales scored. Fused: % scales contacting another scale. Absent: missing scales. (**E**) Spatial distribution of pHH3 positive cells in 4 month-old wild-type and K14ΔNLef1 mutant mice. z-test $p<0.05$ (*). pHH3 cell density was normalised to WT scale. Error bars: S.D. (**I-J**) K31 and CDP (HF marker) immunostaining of tail skin sagittal sections from control (**I**) and 4OHT treated P22 K14ΔNβ-cateninER transgenic mice (**J**). Scale bars = 100 μm.

Supplemental Materials and Methods

Immunolabelling procedures

Antigen retrieval in paraffin sections was carried out by boiling for 15 min in Citrate buffer (pH 6). Permeabilisation was performed using 0.5% Triton X 100 (Sigma) and non-specific antibody binding was blocked using PBS containing 3% BSA and 10% bovine serum. Incubations with primary and fluorescent secondary antibodies were carried out for 2h at room temperature. Slides were mounted using Pro-Long Gold anti-fade reagent (Invitrogen) containing the nuclear counterstain 4′,6-diamidino-2-phenylindole (DAPI).

To prepare wholemounts, skin was incubated in PBS/5 mM EDTA in a 37 °C water bath for 1 to 4 h. The epidermis was then separated from the dermis and fixed

with 2% formaldehyde for 10 minutes. Antibodies were diluted in PB buffer (PBS containing 0.5% milk powder, 0.25% fish skin gelatin and 0.25% Triton X 100) and incubations carried out overnight. GFP immunohistochemistry was performed with an automatic staining system (Leica ST5020, Leica Microsystems; Wetzlar, Germany) and the Bond Intense R Detection kit (Leica Microsystems; Wetzlar, Germany).

Primary antibodies used were: rat anti-pCADHERIN (13-2000Z, Zymed Laboratories), rabbit anti-CASPASE14 (ab1653, Abcam), rabbit anti-CASPASE14 (kind gift from W. Declercq), rabbit anti-CDP (sc-13024, Santa-Cruz), rabbit anti-DCT (BS 3320, Bioworld Technology), rabbit anti-FILAGGRIN (ab24584, Abcam), chicken anti-GFP (ab13970, Abcam), rabbit anti-pHISTONE H3 (9701, Cell Signaling Technology), mouse anti-INVOLUCRIN (SY3, prepared-in-house), mouse anti-KERATIN2e (ab19122, Abcam), mouse anti-KERATIN10 (sc-23877, Santa-Cruz), guinea-pig anti-KERATIN31 (GP-hHa1, Progen), rabbit anti-LEF1 (2230, Cell Signaling Technology), goat anti-LRIG1 (AF3688, R&D Systems), goat anti-SOX9 (AF3075, R&D Systems) and goat anti-SOX10 (sc-17342, Santa-Cruz). Species-specific secondary antibodies conjugated to AlexaFluor 488 or AlexaFluor 555 (Invitrogen) were also used. Polymerised actin was visualised using phalloidin coupled with AlexaFluor 594 (Invitrogen).

Clonal analysis

Scoring GFP-positive clones. The 3-D positions of GFP-positive cells and whether or not they expressed the AB1653 scale marker were determined using image z-stacks of tail epidermal whole mounts. We considered a clone to be any GFP-positive cell or group of cells that were separated from another GFP-positive cell or group of cells by

a distance of at least 3 GFP-negative cells. Only IFE clones were scored. Clones were placed into four categories:

a. Clones outside scales: located entirely in the interscale region and not in contact with scale boundaries.
b. Clones inside scales: located entirely inside the scale region and not in contact with scale boundaries.
c. Clones not crossing scale boundaries: located entirely within either a scale or interscale region, but with at least one cell in direct contact with the boundary.
d. Clones crossing scale boundaries: containing at least one cell in interscale and scale regions.

Observed and expected distribution of clones. A theoretical probability model was developed to investigate whether the observed distribution of GFP clones at P9 was significantly different from that expected if clones are founded by bi-potential progenitors.

First, the distribution of clones at induction was considered. At P2, the clones appeared to be randomly distributed (Figure S2A-D) and considered to reflect their position at P0. From this, it was considered reasonable to assume that keratin 14 was expressed homogenously in the basal layer and that tamoxifen induced GFP with the same probability in each cell. Thus, since the density of clones was low, their distribution could be modelled by a Poisson distribution.

In order to determine if the observed clones at P9 also followed a Poisson distribution (as would be expected if their development is unaffected by any external influences) a directly comparable, theoretical population of clones was constructed based on the observed data. To do this, the average surface areas of scales, clones and

cells (in µm²), as well as the proportion of the total area occupied by scales, were first determined on maximum intensity z-projections of the original z-stacks. From this, the skin was modelled as a flat surface, with scales, clones and cells as circles with a surface area equal to that of the average values obtained, and scales distributed at a density such that the total area they occupied was the same as in the confocal images. These theoretical scales, clones and cells were used to define four regions with analogous definitions to those used to assess the locations of actual clones:

1) Interscale region: all points where the centre of a clone could be located such that the clone would remain entirely outside the scale and no part of it would come within one cell radius of the boundary (pink region in Figure S2G)
2) Scale region: all points where the centre of a clone could be located such that the clone would remain entirely inside the scale and no part of it would come within one cell radius of the boundary (orange regions in Figure S2G)
3) Tangential region: all points where the centre of a clone could be located such that at least part of the edge will come within one cell radius of the boundary, but that the clone does not cross the boundary by more than one cell radius. (blue regions in Figure S2G)
4) Crossing region: all points where the centre of a clone could be located such that the clone crosses the boundary by at least one cell radius (purple region in Figure S2G)

The construction of these regions is shown in Figure S2. First, the theoretical scale (orange) and interscale structures (pink) are drawn (Figure S2E). A ring is extended from both sides of the scale-interscale boundary by one clone radius to create a "tangent and crossing region" (grey, Figure S2F). All clones with a centre falling in this region will have at least part of the edge crossing the boundary (red clones), and

only clones with the centres falling on the edges of the ring, strictly speaking, will be tangential to the boundary (blue clones). However, because the position of a clone relative to a scale by definition cannot be resolved better than one cell diameter, a correction is required to account for this. Both the edges of the grey region were thus extended on both sides by a cell radius to define the tangential region (blue) and the crossing region (purple) (Figure S2G).

As the theoretical clone population is defined as distributed by the Poisson distribution, it follows that the expected proportion of clones in each region is equal to the proportion of the area that it occupies. The observed proportions were then compared to the theoretical proportions using the binomial distribution and the differences assessed for statistical significance.

Scoring the number of cells per clone. Cell number was determined in every clone analysed at P9 by analysing z-stacks. The average cell number per clone was calculated for each area of the IFE and plotted in the graph Figure 2D. The statistical significance of the difference in clone size between areas was assessed using a t-test.

Scoring phospho-histone H3 positive cells

Immunostaining for K31 and phospho-histone H3 was performed on wholemounts of tail epidermis of three wild-type mice at P9 (Figure 2G) and one 4 month-old K14ΔNLef1 and its wild-type littermate (Figure S4E). Images were acquired as z-stacks and phospho-histone H3 positive cells were scored on maximum intensity projections.

At P9, the tangential areas to scale were defined according to the average size of the clones observed by lineage tracing, that is, around 4 cell-diameters from the scale boundary for the internal tangential area and 3 cell-diameters from the scale

boundary for the external tangential area to scale. Hence, any phospho-histone H3 positive cells within 4 cell-diameters from the inside scale boundary were scored as “lining scale inside” and any phospho-histone H3 positive cells within 3 cell-diameters from the outside scale boundary were scored as “lining scale outside”. The rest were scored as “inside” (further than 4 cell-diameters) and “outside” (further than 3 cell-diameters) scales. Each score for each mouse was proportioned to the surface of the four areas (calculated from lineage tracing data). The average of the proportioned values was calculated and plotted on the graph in Figure 2G. A total of 857 phospho-histone H3 cells was scored.

In the wholemounts of epidermis from 4 month-old mice, because of the absence of lineage tracing data, only the total scale and total interscale areas were scored after substracting the area occupied by HF using imageJ. The number of phospho-histone H3 positive cells in scale and interscale was then proportioned to the corresponding measured surfaces of scale and interscale (Figure S4E). A total of 1139 phospho-histone H3 cells (spanning around 160 scales) was scored in K14ΔNLef1 epidermis and 503 (spanning around 107 scales) in wild-type epidermis.

Given the large number of positive cells (at least 100) in each compartment at both time-points, the normal approximation to the Poisson distribution was used to assess the statistical significance of differences between the compartments using a z-test.

Calculation of cell cycle times in tail IFE

We considered that cell proliferation is exponential. We calculated for each area of tail IFE the cell cycle duration as follows:

$$T = 7 / N_b$$

with

T = Mean cell cycle time

7 = Number of days between P2 and P9

N_b = Number of doublings between P2 and P9

$$N_b = (\log N_{c2} - \log N_{c1}) / \log 2$$

N_{c1} = Average number of cells per clones at P2

N_{c2} = Average number of cells per clones at P9

Supplemental Figure 1

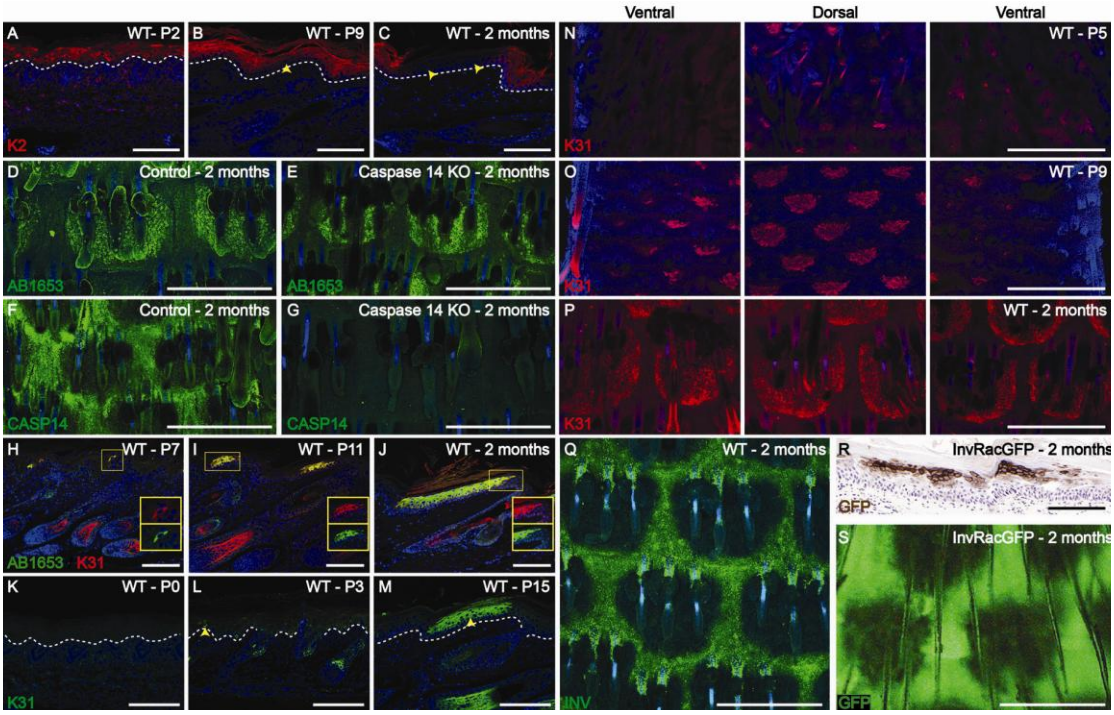

Supplemental Figure 2

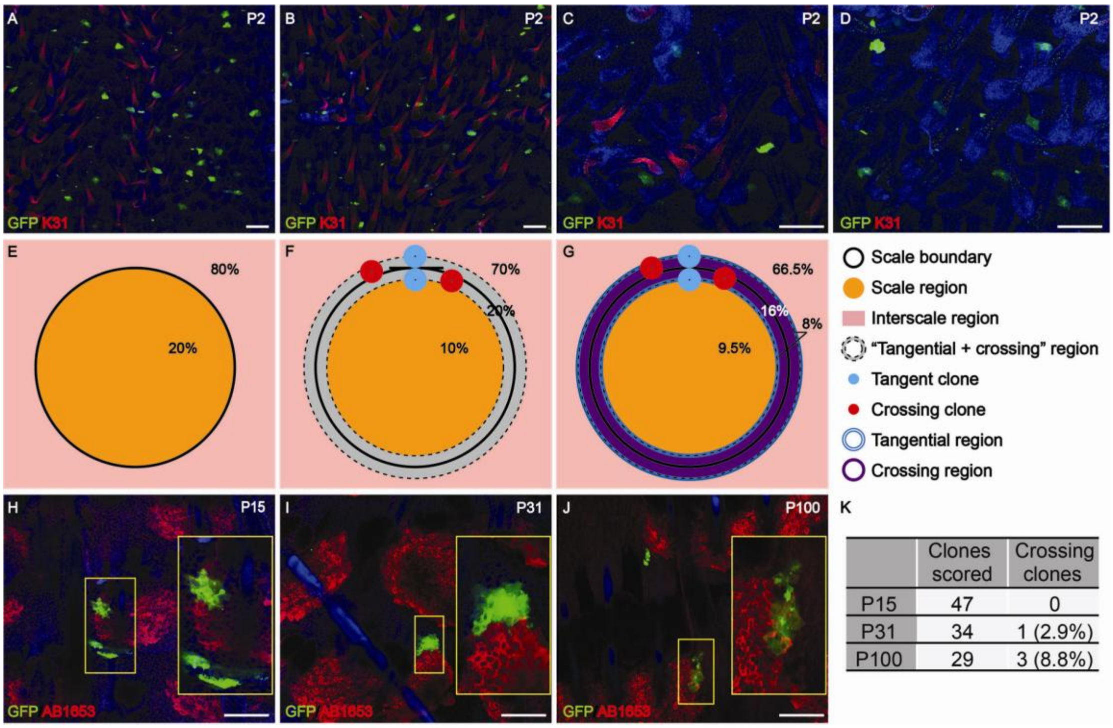

	Clones scored	Crossing clones
P15	47	0
P31	34	1 (2.9%)
P100	29	3 (8.8%)

Supplemental Figure 3

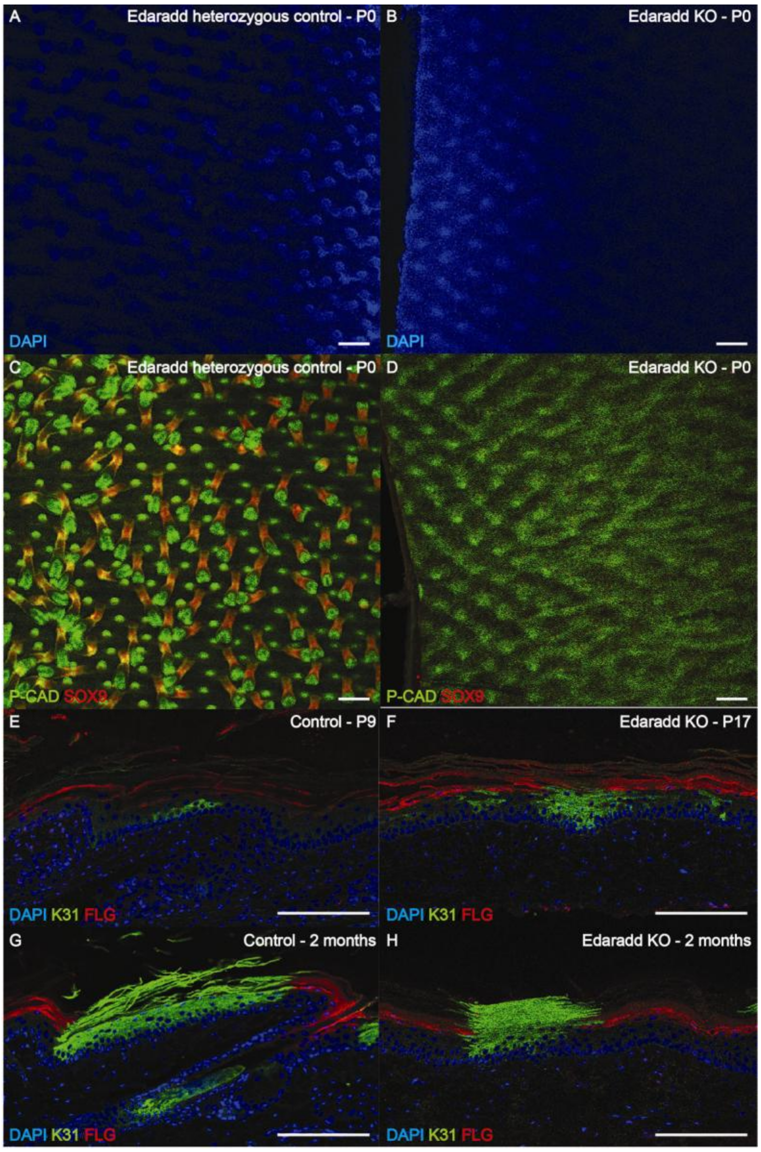

Supplemental Figure 4

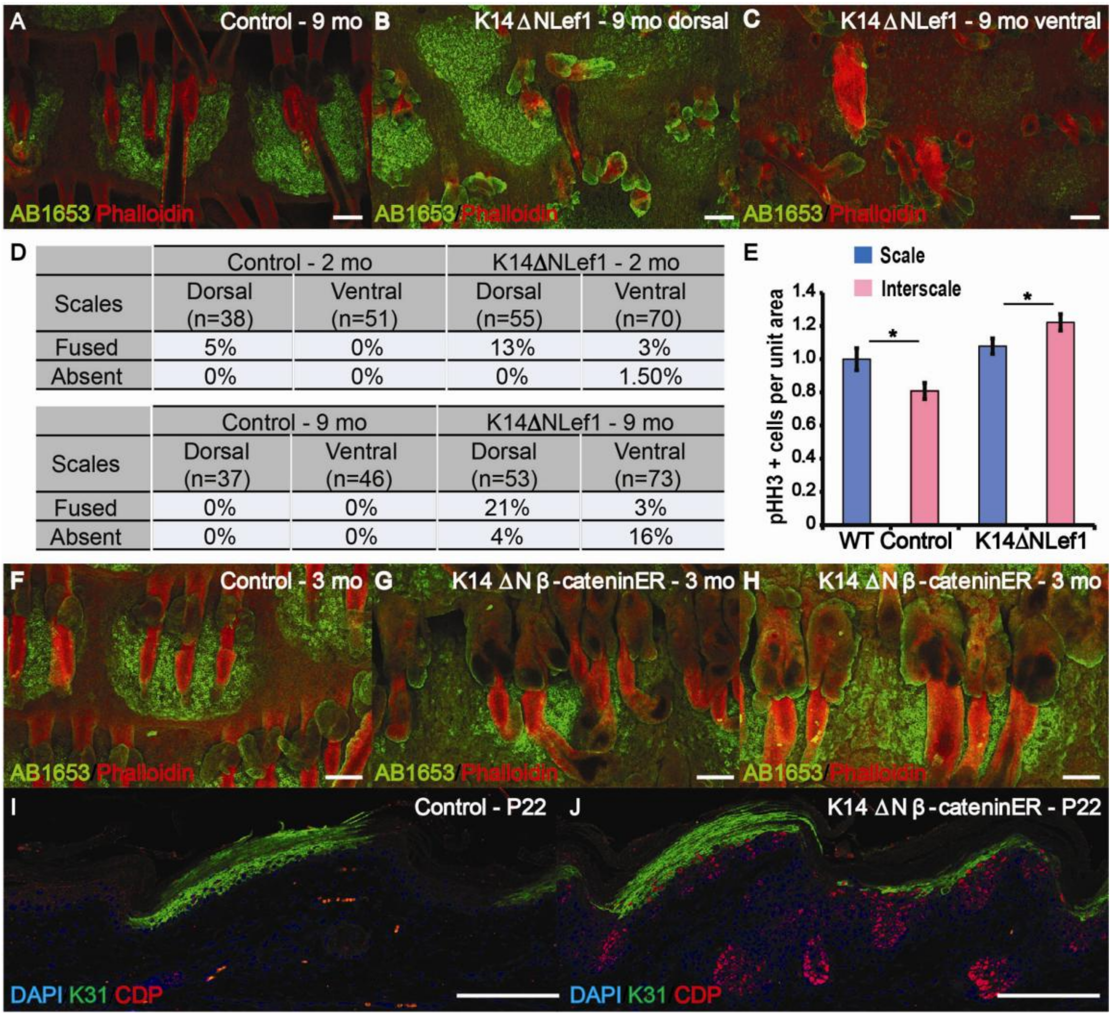

	Control - 2 mo		K14ΔNLef1 - 2 mo	
Scales	Dorsal (n=38)	Ventral (n=51)	Dorsal (n=55)	Ventral (n=70)
Fused	5%	0%	13%	3%
Absent	0%	0%	0%	1.50%

	Control - 9 mo		K14ΔNLef1 - 9 mo	
Scales	Dorsal (n=37)	Ventral (n=46)	Dorsal (n=53)	Ventral (n=73)
Fused	0%	0%	21%	3%
Absent	0%	0%	4%	16%
